# Supplementary material for: Complex‐centric proteome profiling by SEC‐SWATH‐MS
Source: Mol Syst Biol. 2019 Jan 14;15(1):e8438. doi: 10.15252/msb.20188438 (PMC6346213; doi:10.15252/msb.20188438)
Supplement: Supplementary file 8 — Dataset EV7 [file MSB-15-e8438-s008.zip › feature_plots_string/P15735.pdf]

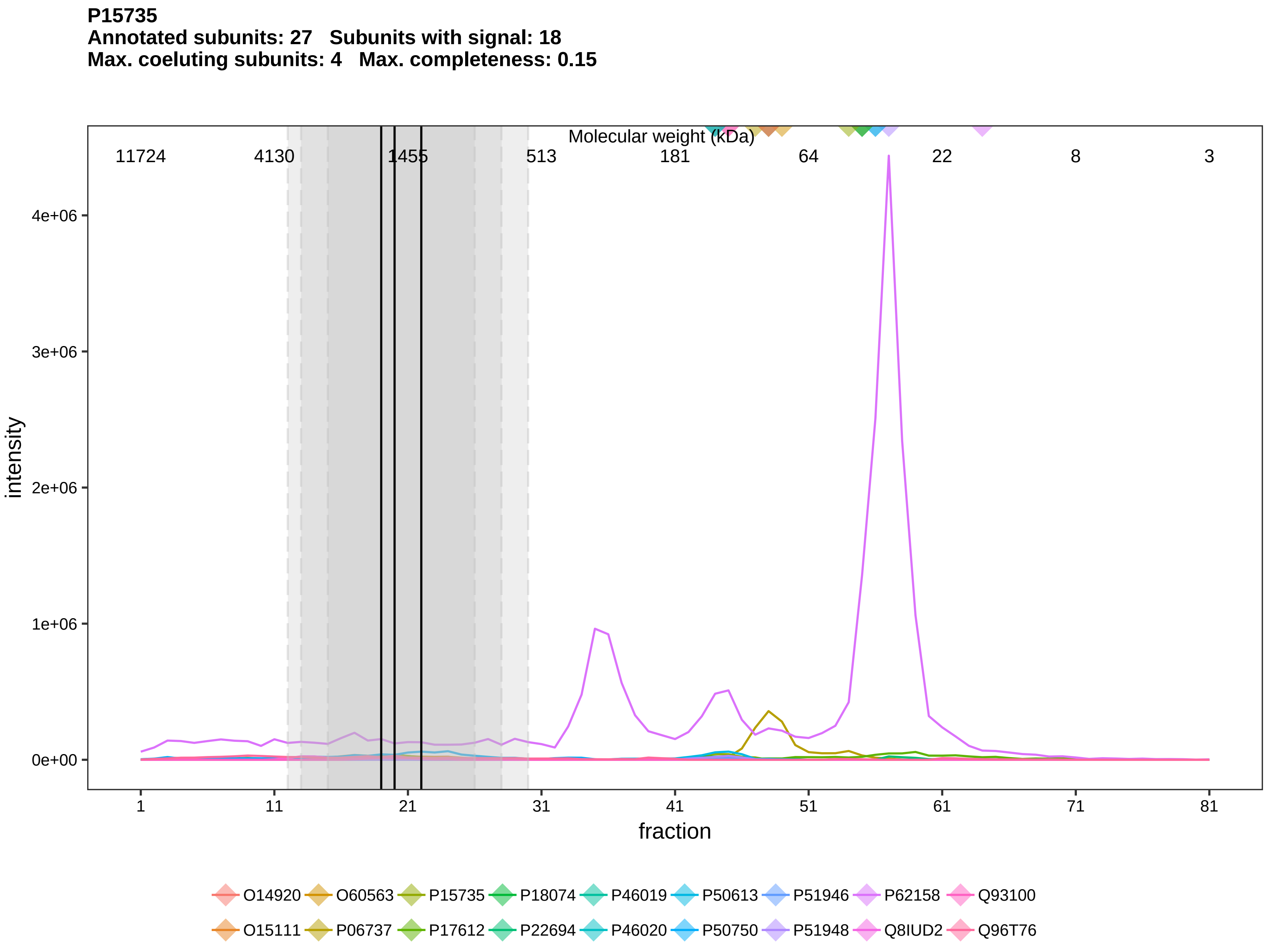
P15735
Annotated subunits: 27 Subunits with signal: 18
Max. coeluting subunits: 4 Max. completeness: 0.15
Molecular weight (kDa)
11724
4130
1455
513
181
64
22
8
3
intensity
4e+06
3e+06
2e+06
1e+06
0e+00
fraction
1
11
21
31
41
51
61
71
81
O14920
O60563
P15735
P18074
P46019
P50613
P51946
P62158
Q93100
O15111
P06737
P17612
P22694
P46020
P50750
P51948
Q8IUD2
Q96T76
